# The complete chloroplast genome sequence of *Nekemias cantoniensis* (Hook. et Arn.) Planch 1873 (Vitaceae)

**DOI:** 10.1080/23802359.2022.2109436

**Published:** 2022-08-26

**Authors:** Yongjian Luo, Ru Wang, Qing Li, Jun Liu, Zhijun Deng

**Affiliations:** a Hubei Key Laboratory of Biologic Resources Protection and Utilization (Hubei Minzu University), Enshi, PR China; b The Plant Germplasm Resources Laboratory, School of Forestry and Horticulture, Hubei Minzu University, Enshi, PR China; c Guangdong Key Laboratory for Crop Germplasm Resources Preservation and Utilization, Agro-biological Gene Research Center, Guangdong Academy of Agricultural Sciences, Guangzhou, China; d Research Center for Germplasm Engineering of Characteristic Plant Resources in Enshi Prefecture (Hubei Minzu University), Enshi, PR China

**Keywords:** Chloroplast genome, small single-copy, phylogenetic relationship, maximum likelihood

## Abstract

*Nekemias cantoniensis* (Hook. et Arn.) Planch 1873 is a woody vine species native to South and Southwest China that is rich in flavonoids and also displays excellent pharmacological activities. The purpose of this study was to characterize the complete chloroplast (cp) genome of *N. cantoniensis* using Illumina pair-end sequencing data. In summary, the complete cp genome of *N. cantoniensis* exhibits a quadripartite structure with a length of 162,655 base pairs, including a large single-copy (LSC) region of 89,341 base pairs, a small single-copy (SSC) region of 19,076 base pairs, and two inverted repeats (IRs) regions of 27,119 base pairs. The overall GC content of the genome is 37.41%, while the corresponding values for the LSC, SSC, and IR regions are 34.75%, 32.89%, and 43.02%, respectively. The genome contains 137 genes, of which 87 are protein coding, 36 are tRNA coding, and eight are rRNA coding. Maximum-likelihood phylogenetic analyses revealed that *N. cantoniensis* was clustered with *N. grossedentata*.

Species of *Nekemias cantoniensis* (Hook. et Arn.) Planch 1873 are woody vines native to South and Southwest China, such as Guangdong, Guangxi, Yunnan, Hainan, and Hong Kong (Editorial Committee of Flora of China 2007). The stem tips and tender leaves of *N. cantoniensis* can be used to make Tengcha tea, which has a health benefit due to its flavonoid content (Gui et al. 2015; Li et al. 2021). For hundreds of years, it has been used to treat colds, fevers, sore throats, gangrenous hepatitis, and boils (Wu et al. 2014). In recent years, pharmacological research has shown that *N. cantoniensis* and its extract have a wide range of pharmacological effects, including antioxidant, antibacterial, liver protection, blood lipid reduction, blood glucose reduction, anti-inflammatory, analgesic, and anti-tumor effects (Gao et al. 2017). The basis for these pharmacological effects is believed to be primarily the presence of secondary metabolites in *N. cantoniensis*, primarily flavonoids, such as dihydromyricetin, myricetin, myricetin, etc. (Chen et al. 2019). In this study, high-throughput sequencing technology was used to sequence, assemble and annotate the complete chloroplast (cp) genome of *N. cantoniensis*. The structural characteristics of *N. cantoniensis* cp genome were statistically analyzed, and the genetic relationship between *N. cantoniensis* and some genera of Vitaceae was accurately located, providing a theoretical basis for the conservation and sustainable utilization of *N. cantoniensis* resources.

The fresh leaves of *N. cantoniensis* were collected from Guangzhou, Guangdong, China (113°45′E, 23°40′N, altitude: 139 m) (Figure 1(A,B)). The scientific committee of the Guangdong Academy of Agricultural Sciences approved the procedure for plant collection. A specimen was deposited at the Guangdong of Agro-biological Gene Research Center (http://multi-omics.agrogene.ac.cn/, contact person: Yongjian Luo, and email:851022933@qq.com) under the voucher number 20210303001. Total genomic DNA was extracted from fresh young leaves of *N. cantoniensis* using a modified CTAB method and quantified according to Allen et al. (2006). We constructed paired-end libraries with insert sizes of 150 bp (our study protocol was approved by the ethics review board of Hubei Minzu University, exemption number 2022031055). Beijing Microread Inc. (Beijing, China) sequenced the complete genome of *N. cantoniensis* using the Illumina HiSeq 2500 platform, resulting in 3.43 Gb of raw reads. SRR16608263 is the project accession number for the raw sequence data deposited in NCBI SRA. According to Bolger et al. (2014), the raw data were then filtered using Trimmomatic Version 0.38 with default settings. GetOrganelle was used to assemble the complete genome of cp according to Jin et al. (2020) and annotated by CPGAVAS2 (http://www.herbalgenomics.org/cpgavas2) according to Shi et al. (2019). A genome annotation for each of the cp species was submitted to GenBank (OK662571) after being reviewed and adjusted manually.

**Figure 1.**
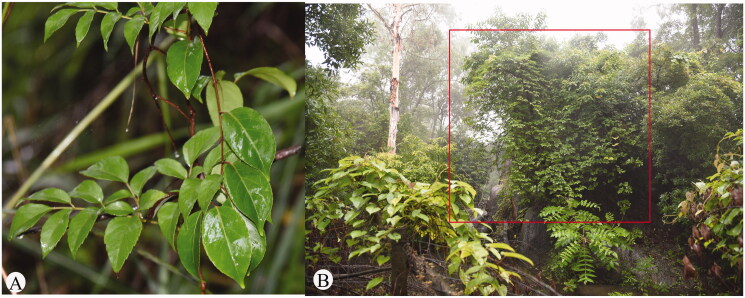
Species reference image of *N. cantoniensis*. (A) Morphological characteristics of leaves of *N. cantoniensis*. (B) The growing environment of *N. cantoniensis* (the ones in the box are *N. cantoniensis*).

The whole cp genome of *N. cantoniensis* was 162,655 bp in length and demonstrated a typical angiosperm circular cp structure, with a large single-copy (LSC) region of 89,341 bp, a small single-copy (SSC) region of 19,076 bp, and a pair of inverted repeat (IR) regions of 54,238 bp (Figure 2). The total GC content of the genome of *N. cantoniensis* cp is 37.41% (LSC, 34.75%; SSC, 32.89%; IR, 43.02%). GC content and gene order were comparable to those of *Nekemias grossedentata* (Gu et al. 2020). There were 131 genes in the cp genome, including 87 protein-coding genes, 36 tRNA genes, and eight rRNA genes. There were introns in 12 protein-coding genes (*rps*16, *rps*12, *rpo*C1, *rp*l2, *rpl*16, *pet*D, *pet*B, *paf*I, *ndh*B, *ndh*A, *clpP*1, and *atp*F) and six tRNA genes (*trn*K-UUU, *trn*R-UCC, *trn*L-UAA, *trn*V-UAC, *trn*I-GAU, and *trn*A-UGC). Sixty-eight SSRs were identified, 61 of which were mono-nucleotides (A/C/T, 88.52%), eight were di-nucleotides (AT/TA, 9.84%), and one was a tetra-nucleotide repeat (TTAA, 1.64%) (Figure 3).

**Figure 2.**
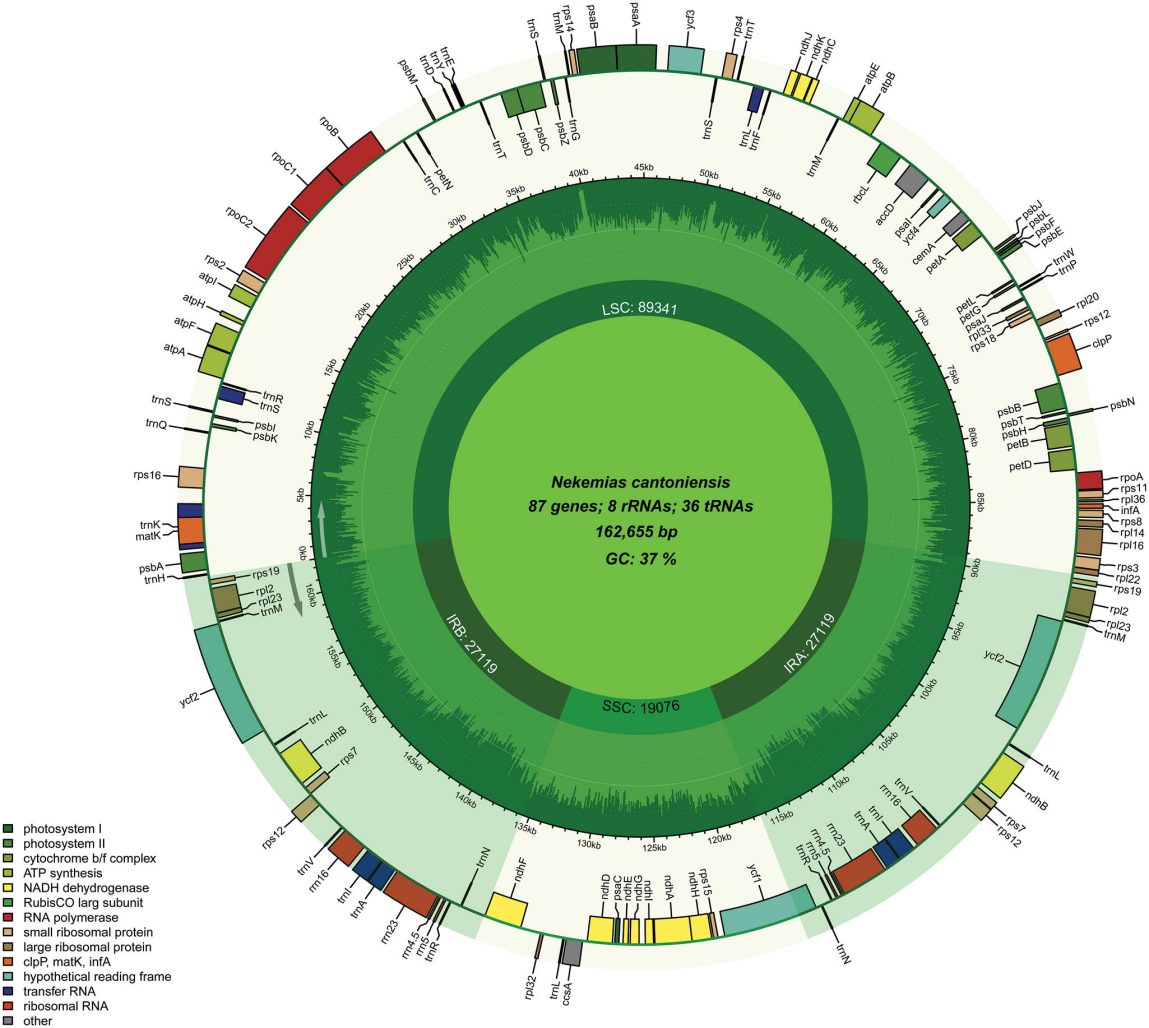
Gene map of chloroplast genome of *N. cantoniensis*. Genes outside the circle are transcribed in counterclockwise direction and those inside in clockwise direction. LSC: large single-copy; SSC: small single-copy; IR: inverted repeat.

**Figure 3.**
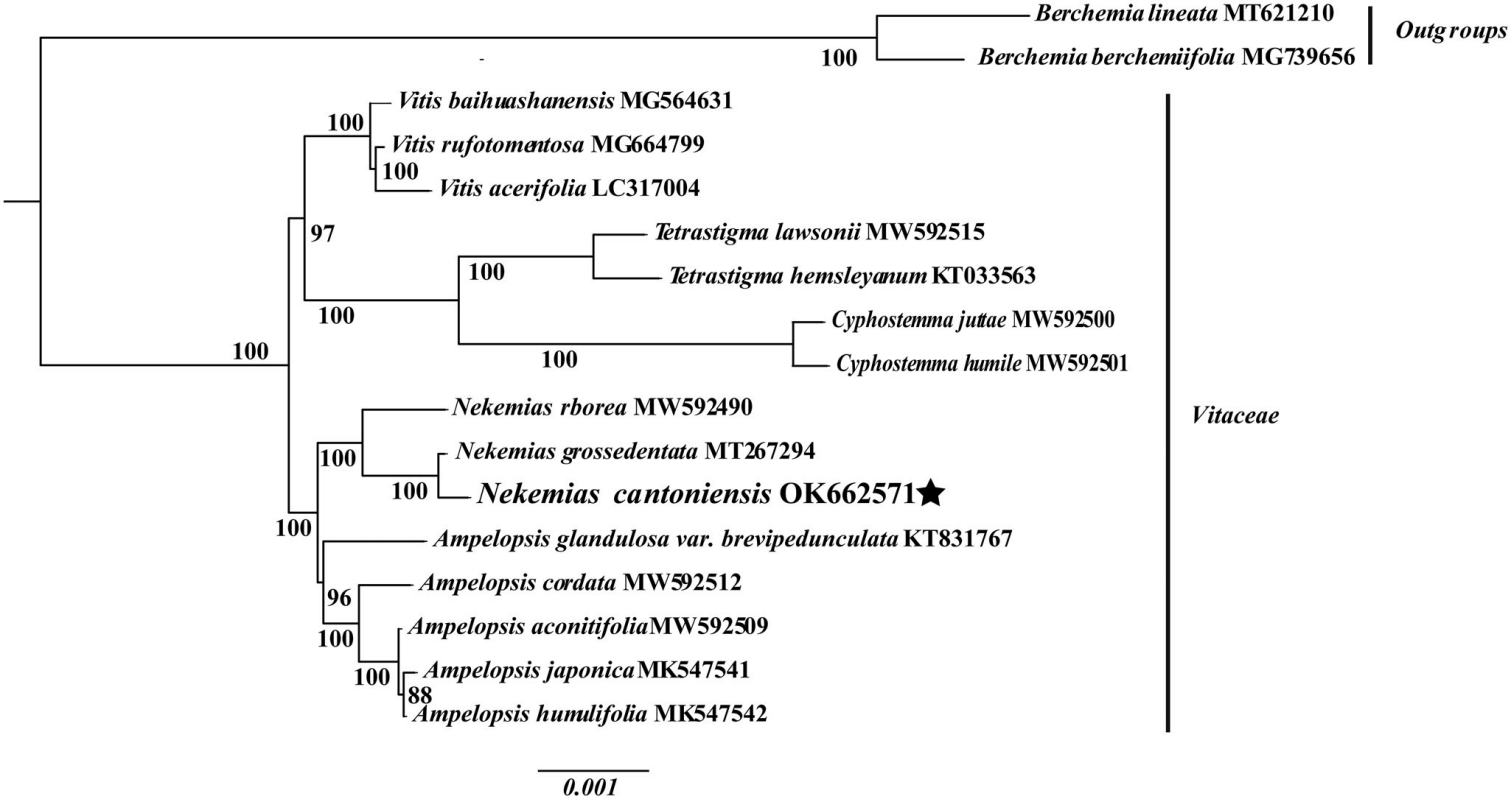
The ML phylogenetic tree based on the complete genome sequences of *N. cantoniensis* and 16 other species.

To confirm the phylogenetic position of *N. cantoniensis* within the Vitaceae family, we generated a maximum-likelihood (ML) phylogenetic tree using 17 cp genomes from the GenBank collection. Using MAFFT V7.309, several sequence alignments were done (Katoh and Standley 2013). A ML phylogenetic tree was generated based on a data matrix of a concatenation of 76 protein-coding sequences, implemented with RAxML v8 (Nguyen et al. 2015).The results confirmed that *N. cantoniensis* was clustered with *N. grossedentata*. This study extends our comprehension of cp genome evolution in *Nekemias*.

## Author contributions

In this research, Yongjian Luo is the experimental designer and executor. He has completed the data analysis and the first draft of the paper. Ru Wang and Qing Li have contributed to the experimental design and the analysis of experimental results. Jun Liu and Zhijun Deng have been responsible for supervising the experimental design, data analysis, and the writing and revision of the paper. The final version of the manuscript was read and approved by all authors.

## Data Availability

In support of the findings of this study, the genome sequence data are openly available in GenBank of the NCBI at https://www.ncbi.nlm.nih.gov/ under accession no. OK662571. Specifically, the associated BioProject, SRA, and Bio-Sample numbers are PRJNA775800, SRR16608263, and SAMN22627476, respectively.
